# AffyMiner: mining differentially expressed genes and biological knowledge in GeneChip microarray data

**DOI:** 10.1186/1471-2105-7-S4-S26

**Published:** 2006-12-12

**Authors:** Guoqing Lu, The V Nguyen, Yuannan Xia, Michael Fromm

**Affiliations:** 1 Department of Biology, University of Nebraska, Omaha, NE 68182, USA; 2 Center for Biotechnology, University of Nebraska, Lincoln, NE 68588, USA

## Abstract

**Background:**

DNA microarrays are a powerful tool for monitoring the expression of tens of thousands of genes simultaneously. With the advance of microarray technology, the challenge issue becomes how to analyze a large amount of microarray data and make biological sense of them. Affymetrix GeneChips are widely used microarrays, where a variety of statistical algorithms have been explored and used for detecting significant genes in the experiment. These methods rely solely on the quantitative data, i.e., signal intensity; however, qualitative data are also important parameters in detecting differentially expressed genes.

**Results:**

AffyMiner is a tool developed for detecting differentially expressed genes in Affymetrix GeneChip microarray data and for associating gene annotation and gene ontology information with the genes detected. AffyMiner consists of the functional modules, *GeneFinder *for detecting significant genes in a treatment versus control experiment and *GOTree *for mapping genes of interest onto the Gene Ontology (GO) space; and interfaces to run Cluster, a program for clustering analysis, and GenMAPP, a program for pathway analysis. AffyMiner has been used for analyzing the GeneChip data and the results were presented in several publications.

**Conclusion:**

AffyMiner fills an important gap in finding differentially expressed genes in Affymetrix GeneChip microarray data. AffyMiner effectively deals with multiple replicates in the experiment and takes into account both quantitative and qualitative data in identifying significant genes. AffyMiner reduces the time and effort needed to compare data from multiple arrays and to interpret the possible biological implications associated with significant changes in a gene's expression.

## Background

DNA microarrays are a powerful tool for monitoring the expression of tens of thousands of genes simultaneously [1]. Affymetrix GeneChips are widely used microarrays with a collection of 11 – 20 probe pairs called a probe set that measures the expression of each transcript. The probe pairs comprise a perfect match (PM) and a single base mismatch (MM) to the target mRNA region.

GeneChip microarrays use a statistical algorithm in the Microarray Suite 5.0 (MAS 5.0; Affymetrix) to estimate the variance among probe pairs within a probe set and to compute an expression index that represents transcript abundance [2]. The MAS 5.0 algorithm uses the One-Step Tukey's Biweight Estimate to compute the Signal intensity for each probe set, and employs the Wilcoxon signed-rank test to assess the Detection calls and p-values for a single array analysis [3,4]. The algorithm uses normalization and scaling techniques to correct for variations between two arrays [5]. The comparison analysis of two arrays results in data matrices such as Change p-value, Change, and Signal Log Ratio. In the case of replicate sample analysis, the two sample statistical tests such as the Student t-test or the Mann-Whitney test can be used to test the hypothesis whether the signal intensity values for each probe set are significantly different in the treatment group compared with the control group. Such statistical tests are not ideal for finding significant genes, because only a few replicate samples (< 4) are usually used in the microarray experiments. Determining the most appropriate statistical method for detecting differentially expressed genes in GeneChip replicate data remains a challenging issue.

Several methods have been developed to improve the sensitivity and selectivity for detecting significant genes in GeneChip microarray experiments. The widely used algorithms include the robust multiarray average (RMA) [6], the model based expression index/intensity (MBEI) implemented in dCHIP software [7], and the positional dependent nearest-neighbor model (PDNN) [8]. These algorithms effectively deal with the 'probe effect', that is, some probes in a probe set tend to give higher values than others [2], through re-computing of the signal intensity for each probe set using the processed image data exported from Affymetrix Microarray Suite or GeneChip Operating Software (GCOS). These methods rely solely on the quantitative data, i.e., signal intensity for the comparison analysis. However, qualitative data such as Detection call are also important parameters in detecting significant genes. Using a threshold fraction of Present detection calls can ultimately eliminate the unreliable probe sets while preserving the most significant ones [9]. A combination of a qualitative parameter (change call) and two quantitative parameters (fold change and signal mean ratios) reduces greatly the false positives, while using a single parameter has a greater than 30% false positive rate [10].

Here we present a software tool called AffyMiner that uses both the quantitative and the qualitative data metrics for detecting differentially expressed genes in GeneChip data. In addition, AffyMiner has functions for connecting gene annotation information and Gene Ontology (GO) descriptions to the detected significant genes for better biological interpretation of the results.

## Implementation

### Software Design

#### User requirements

These requirements were established from discussions with the users of our Microarray Core Facility over the past three years.

• Compatibility with the data formats exported from Affymetrix MAS or GCOS. The exported data contain Probe sets, Signal detection, Signal value, Signal log ratio, Change, Change *p-value*, etc.

• Provide the user the flexibility choosing different data metrics and different threshold values for filtering for differentially expressed genes.

• Incorporate statistical analysis for the selection of significant genes.

• Facilitate exploratory analyses such as clustering analysis.

• Incorporate information from Gene Ontology and metabolic pathways.

• Have easy-to-use graphical interfaces and provide ready-to-publish charts and tables.

#### Architecture

**Figure 1:**
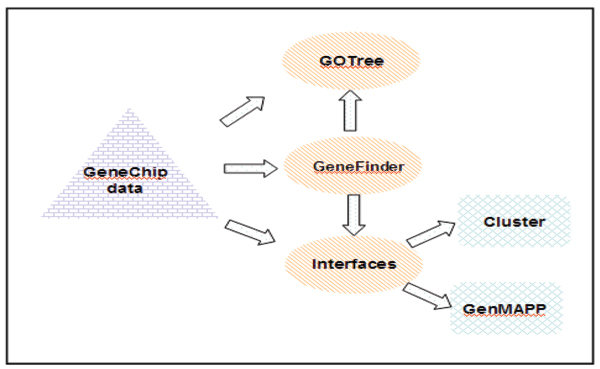
**The Architecture of AffyMiner**. AffyMiner includes two functional modules, *GeneFinder *and *GOTree*, and interfaces to third-part programs.

Based upon the user requirements and our experience in using commercial and open source microarray analysis software packages such as GeneSpring [11] and Bioconductor [12], we designed AffyMiner to include two functional modules, *GeneFinder *and *GOTree*, and interfaces to third-part programs (Figure 1). These modules can analyze GeneChip data separately or consecutively. For example, the gene list generated by *GeneFinder *can be used by *GOTree*. Two popular open source software programs, Cluster and GenMAPP, were chosen for clustering and pathway analysis, respectively.

### Algorithms

#### GeneFinder

**Figure 2:**
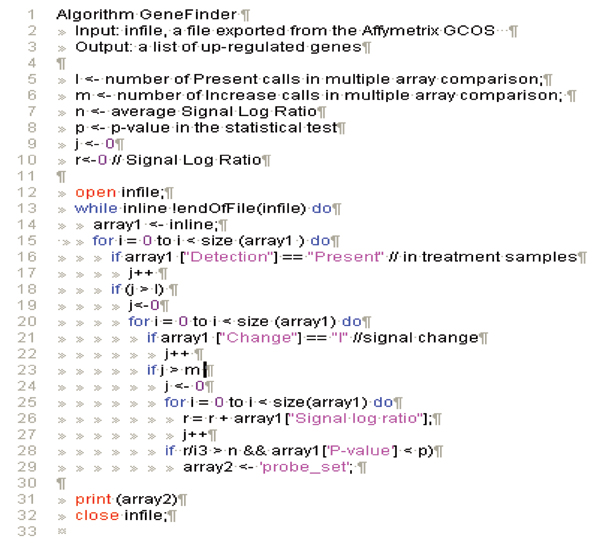
**The algorithm for detecting up-regulated genes**. The high level algorithm involves the following procedures: 1) eliminate the probe sets with signal Detection calls of "Absent" in the treatment samples; 2) select the probe sets with signal Change calls of "Increase"; 3) eliminate the probe sets with a Signal Log Ratio below a threshold defined by the user; and 4) remove the probe sets with a p-value above a threshold defined by the user.

The algorithm implemented in *GeneFinder *uses both the qualitative and quantitative measures of transcript performance, including Detection, Change, Signal Log ratio, and the statistical results. To determine significantly up-regulated genes in an experiment with multiple replicates of treatment and control samples the following steps are used: 1) eliminate the probe sets with signal Detection calls of "Absent" in the treatment samples; 2) select the probe sets with signal Change calls of "Increase"; 3) eliminate the probe sets with a Signal Log Ratio below a threshold defined by the user; and 4) remove the probe sets with a *p*-value above a threshold defined by the user (Figure 2).

The algorithm for detecting significantly down-regulated genes is as follows: 1) eliminate the probe sets with signal Detection calls of "Absent" in the control samples; 2) select the probe sets with signal Change calls of "Decrease"; 3) eliminate the probe sets with a Signal Log Ratio above a threshold defined by the user; and 4) remove the probe sets with the *p*-value above a threshold defined by the user.

#### GOTree

The Gene Ontology (GO) Consortium produces structures of biological knowledge using a controlled vocabulary consisting of GO terms [13]. GO terms are organized into three general categories, biological process, molecular function, and cellular component. The terms within each category are linked in defined parent-child relationships that reflect current biological knowledge. All genes from different organisms are systematically associated with the GO terms, and these associations continue to grow in complexity and details as sequence databases and experimental knowledge grow [14]. GO provides a useful tool to look for common features shared within a list of genes.

The high-level description of the algorithm in building the GO tree is as follows, 1) read the output file generated by *GeneFinder*; 2) write in an array the GO IDs and their corresponding Affymetix probe set IDs; 3) find the GO Path IDs for each GO ID in the array and add the GO Path IDs to each element in the array; 4) sort by the GO Path IDs and compute the sum of the probe sets associated with each node; 5) build the entire tree based on the GO Path IDs and write in each node the GO term, GO ID, and the number of probe sets.

#### Programming

AffyMiner was programmed in Visual Basic (VB) .Net on the Microsoft .Net platform. VB .Net is the latest version of the Microsoft Visual Basic language. It has many attractive features, such as easy of use, fully object-oriented, and true visual development [15].

## Results

**Figure 3:**
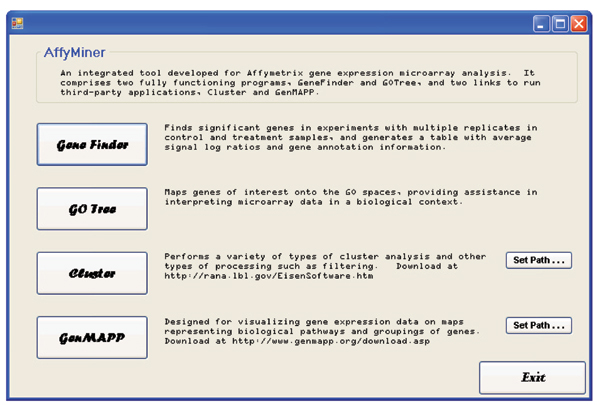
**The main window of AffyMiner**. It consists of brief descriptions of AffyMiner and its two modules, *GeneFinder *and *GOTree*, and interfaces to Cluster and GenMAPP.

AffyMiner includes *GeneFinder*, *GOTree*, and *Interfaces *to Cluster and GenMAPP as shown in the main window of AffyMiner (Figure 3). The brief descriptions of AffyMiner and its modules are available in this window. To illustrate the functions of AffyMiner, we will use Affymetrix *Drosophila *Genome 2.0 array data, produced in the aging experiment with caloric restricted *Drosophila*, where there are 3 control replicates and 3 treatment replicates for each time point.

### GeneFinder

*GeneFinder *has two programs: *Significant Genes *for finding differentially expressed genes satisfying the user defined criteria, and *Annotation *for linking gene annotation information with the gene list.

### Significant Genes

The *Significant Genes *program has interactive interfaces to set up parameters, upload input files, and define the output, respectively. The parameter-setting window contains three frames for setting up the number of replicates, the direction of a robust change, and the data metrics for detecting differentially expressed genes. AffyMiner limits the maximum number of replicates to five. This is a reasonable assumption because the reproducibility of Affymetrix GeneChip array data is high and most publications use two to three replicates in their experiments. The data metrics consist of Signal Detection, Signal Change, Signal Log Ratio and Statistical Test. The user can choose the data matrices and threshold values for each analysis.

**Figure 4:**
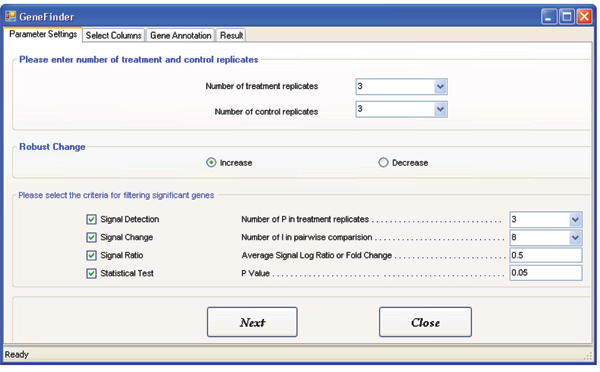
**Parameter setting window. **In this window, there are three frames for parameter settings, including the number of treatment and control replicates, change direction (i.e., Increase or Decrease), the criteria for filtering significant genes.

As shown in Figure 4, three treatment replicates and three control replicates were used for example analysis. The radio button Increase was checked, which means finding significantly up-regulated genes. In the frame "Please select the criteria for filtering significant genes", the signal detection level was set to 3, meaning the Present calls in the signal detention value are required to be present in all the 3 treatment replicates. The number of the signal Change calls was set to 8, which means that at least 8 Increases are required in the 9 Change calls for any given probe set considered significant. The threshold for average signal log ratio was set to be 0.5, which requires about a 1.4-fold increase of the signals in the treatment samples compared with the control samples. The *p*-value for the statistical significance was set to be 0.05. The above settings can be changed dynamically.

**Figure 5:**
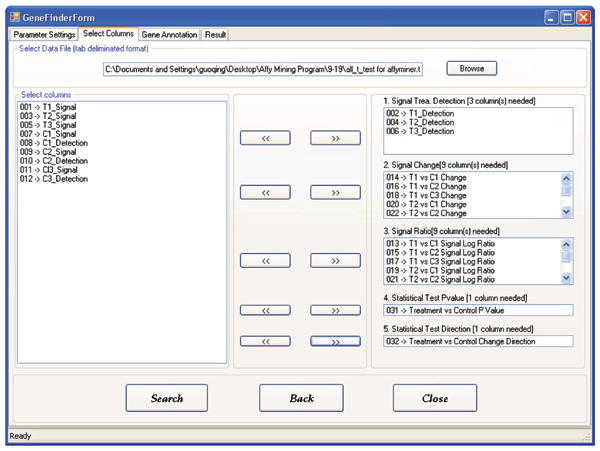
**Input setting window**. Through this window, we can upload the input data file and select columns corresponding to specific samples (i.e., treatment and control) and data metrics. The input file is a text file exported from Affymetrix GCOS.

**Figure 6:**
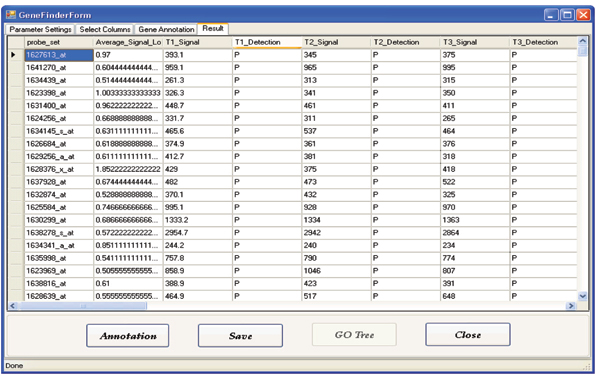
**Output window of the *Significant Genes *program **It shows differentially expressed genes found by the *Significant Genes *program. Note that average signal log ratios are computed and automatically added in the output (i.e., the second column in the output table).

The next step is to upload the input file and select columns corresponding to specific samples (i.e., treatment and control) and data metrics (Figure 5). The input file is a text file exported from Affymetrix GCOS, containing the results of single array analyses and pairwise array comparison analyses. If the Significant Test box was checked in the parameter setting window (Figure 4), the result of the statistical tests or other analysis methods such as RMA need to be added to the text file with two columns corresponding to the *p*-values and the change direction, respectively. The change direction is specified by the "up" in the field of change direction for the up-regulated gene and "down" for down-regulated genes. Clicking the "Back" button returns to the first window if the parameter settings need to be changed. Clicking the "Search" button starts the analysis process. Figure 6 shows the significant genes found by the *Significant Genes *program in *GeneFinder*.

### Annotation

The *Annotation *program links the annotation information with gene lists, and generates a user-defined table with quantitative data such as signal log ratio and qualitative data such as annotation information. The NetAffx annotation file needs to be in the CSV (Comma Separated Value) format, which can be downloaded from the Affymetrix website [16].

The input file for the gene list can be the result generated by *Significant Genes *or any text file with a column corresponding to Affymetrix probe set IDs. Once these two files are uploaded, the data items in the output table can be chosen from the left list box. If not ideal, the user can remove the selected items from the right list box, which will not be shown in the output table.

**Figure 7:**
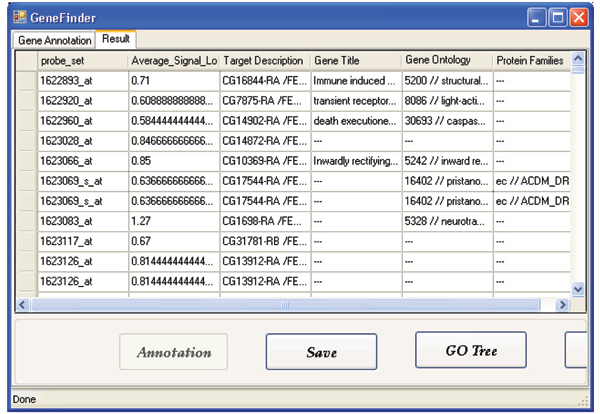
**Output window of the *Annotation *program**. This window shows annotation results generated by the *Annotation *program, where Average Signal Log Ratio, Target Description, Gene Title, Gene Ontology, and Protein family were selected.

The table resulting from the *Annotation *program is shown in Figure 7, where Average Signal Log Ratio, Target Description, Gene Title, Gene Ontology, and Protein family were selected.

### GOTree

**Figure 8:**
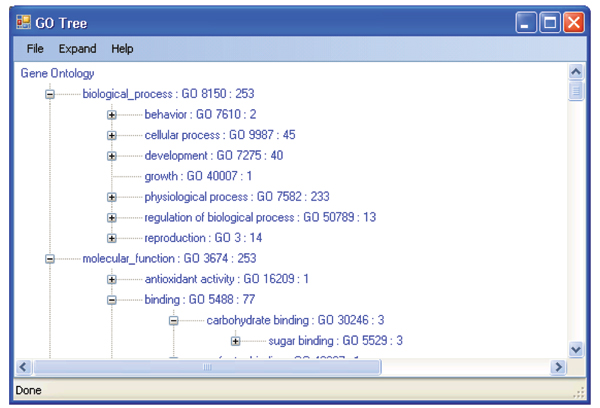
**Output window of the *GOTree *program**. In the tree, each node is labeled with the corresponding GO term, GO ID, and the number of genes associated. The tree can be expanded or clipped by simply clicking on the small square boxes.

*GOTree *takes as input two files. The first file called GOPath consists of the information about the hierarchical structure of GO terms, whereas the second file contains the list of significant genes and their GO term associations. The GOPath file was generated from the *ChipInfo *program, which can be downloaded from the Web [17]. To run *ChipInfo*, the gene information file downloaded from the Affymetrix website is required. The GO tree generated from AffyMiner is shown in Figure 8. Each node is labeled with the corresponding GO term, GO ID, and the number of genes associated. For example, line 3 of the Gene Ontology tree as shown in Figure 8 indicates the node represents behavior in biological process with GO ID 7610 and 2 probe sets on the significant gene list associated with this GO term. The tree can be expanded or clipped by clicking on the small square boxes. A window displaying the Affymetrix IDs associated with the GO term will pop up when the number is right clicked.

### Interfaces to Cluster and GenMAPP

Both Cluster and GenMAPP programs need to be downloaded and installed on the local computer (see below for system requirements of the computer). Go to the websites,  and  to download Cluster and GenMAPP, respectively. In the main window, clicking the button "Set Path ..." will set up the path to the corresponding program file (Figure 1). Clicking the button Cluster or GenMAPP will run the program for analysis.

### Applications

AffyMiner has been tested by multiple users and their feedback has been incorporated into its current version. Results analyzed by AffyMiner have been presented in several publications [18,19]. In the following example, we describe a case study using AffyMiner to compare the lists of differentially expressed genes detected by AffyMiner and the RMA method.

Our group (M. Fromm and Y. Xia) studied the gene expression changes in the retroperitoneal white adipose tissue (RP-WAT) in mice fed trans-10, cis-12 conjugated linoleic acid (t10c12 CLA) [20]. The Affymetrix Mouse Genome 430 2.0 microarrays were used to detect the expression changes of about 34,000 transcripts. Mice were sampled 1, 2, 3, 4, 7, 10, or 17 days after being fed control or 0.5% t10c12 CLA diets, generating 7 time points in total. At each time point, the RP-WAT tissues of ten control and ten t10c12 CLA-fed mice were harvested in groups of five mice each to provide two control and two treatment samples for microarray analysis.

To detect differentially expressed genes the transformed RMA expression values were analyzed using an empirical Bayes Linear model [21,22]. A total of 5407 genes were found significant on Day 1 by the RMA approach. We used the same dataset and ran AffyMiner with the following parameter settings: for increase, 2 Present calls in the treatment samples, 3 Increase calls for signal Change, average signal Log Ratio being 0.5; for decrease, 2 Present calls in the control samples, 3 Decrease calls for signal Change, average signal Log Ratio being -0.5. AffyMiner found 4089 differentially expressed genes. The number of overlap genes found by AffyMiner and RMA is 2946 (Table 1). Moreover, all seven genes validated by the quantitative RT-PCR were found by AffyMiner as well as RMA [20].

## Discussion

Microarray technology has revolutionized the analysis of gene expression. The challenge associated with this high throughput technology is the statistical analysis and biological interpretation of microarray data. AffyMiner was developed to address these issues through finding genes with significant changes in gene expression, and linking these genes with the annotation and Gene Ontology information. Functionally, AffyMiner has overlap with other existing programs, but has the distinguishing features discussed below.

Affymetrix Data Mining Tool (DMT) can filter genes of interest based on the thresholds of certain quantitative and qualitative parameters, but not as powerful as AffyMiner in this aspect. AffyMiner takes full advantage of the range of the different data metrics available from MAS 5.0. AffyMiner provides the flexibility to choose different data metrics (Signal Detection, Signal Change, Signal Log Ratio, and Statistic Test) and to set threshold values for analyzing differentially expressed genes. This flexibility is very important since there is not a single analysis method that outperforms other methods of analyzing microarray data [23,24]. It is evident from the different gene lists generated by AffyMiner and the RMA based approach. Incorporating the qualitative data metrics such as Detection and Signal Change would increase the selectivity of detecting differentially expressed genes [24,25].

GenePicker has certain functions similar to those in AffyMiner [10]. GenePicker was developed for the analysis of replicates of Affymetrix gene expression microarrays. The GenePicker analysis is done through defining analysis schemes, data normalization, t-test/ANOVA, and Change-fold Chang-analysis, and the use of Change Call, Fold Change, and Signal mean ratios. GenePicker provides a comparison of noise and signal analysis scheme for determining a signal-to-noise ratio in a given experiment, which is not available in *GeneFinder*. However, *GeneFinder *uses one more data matrix, i.e., Detection. As mentioned earlier, *GeneFinder *also has the function of incorporating gene annotation information with expression data, which is not available in GenePicker.

The Affymetrix NetAffx Gene Ontology Mining Tool can create a graph of GO terms associated with the input probe sets. However, the graph is very difficult to read as compared with the one generated by AffyMiner (Figure 7). AffyMiner has the flexibility of displaying the GO tree at different levels and the probe sets associated with the GO terms can be viewed easily. Another GO tool called GoSurfer was developed for the GO analysis of Affymetrix GeneChip data [7,14,17]. GoSurfer associates user input gene lists with GO terms and visualizes such GO terms as a hierarchical tree. GoSurfer compares two lists of genes in order to find which GO terms are enriched in one list of genes but relatively depleted in another. GoSurfer can not map genes from a single list onto the GO descriptions. In this regard, *GOTree *and GoSurfer complement each other in the analysis of Gene Ontology.

## Conclusion

As a whole, AffyMiner fills an important gap in finding differentially expressed genes from Affymetrix GeneChip microarray data. AffyMiner effectively deals with multiple replicates in the experiment, provides users flexibility choosing different data metrics for detecting significant genes, and is capable of incorporating various gene annotations. AffyMiner has been used for analyzing the GeneChip data for several publications, which has reduced the time and effort needed to compare data from multiple arrays and to interpret the possible biological implications associated with significant changes in a gene's expression.

## Availability and requirements

• Project name: AffyMiner project

• Project home page: http://bioinfo-srv1.awh.unomaha.edu/affyminer/[26]

• Operating system(s): Microsoft Windows 2000 or later

• Programming language: Visual Basic .Net.

• Other requirements: .NET Framework 2.0 or later. It can be downloaded from our website [26] or Microsoft website [27].

• Installation: To install AffyMiner, double click on AffyMinerInstaller.msi and follow the instructions.

• Any restrictions to use by non-academics: yes, contact the author GL for details.

## Authors' contributions

GL conceived of the study, participated in its design and coordination, and drafted the manuscript. TN carried out the implementation. YX participated in the design and testing and helped to draft the manuscript. MF helped to draft the manuscript.

## Figures and Tables

**Table 1:** Differentially expressed genes detected by approaches of AffyMiner and RMA

**Approach**	**Genes**	
	
	**Up-regulated**	**Down-regulated**
AffyMiner	1927	2162
RMA/Bayesian approach	2530	2877
Common in both	1432	1514
